# Clinical and Instrument-Based Evaluation of Plasma IQ Microcurrent Radiofrequency for Periorbital Skin Rejuvenation

**DOI:** 10.3390/biomedicines14030679

**Published:** 2026-03-16

**Authors:** Paweł Kubik, Wojciech Gruszczyński, Aleksandra Pawłowska, Maciej Malinowski, Brygida Baran, Agnieszka Pawłowska-Kubik, Łukasz Kodłubański, Bartłomiej Łukasik

**Affiliations:** 1 K-LAB Badania i Rozwój, 81-312 Gdynia, Poland; wojciech.gruszczynski@k-lab.com.pl (W.G.); aleksandra.pawlowska@k-lab.com.pl (A.P.); maciejmal@gmail.com (M.M.); agnieszkapawl@wp.pl (A.P.-K.); 2 Medical Department, Matex Lab Switzerland SA, 1228 Geneve, Switzerland; brygida.baran@neauvia.com (B.B.); bartlomiej.lukasik@neauvia.com (B.Ł.); 3 Department of Human Rights and Intellectual Property Law, University of Gdansk, 80-309 Gdansk, Poland; lukasz.kod@gmail.com

**Keywords:** microcurrent radio frequency therapy, skin laxity, Plasma IQ, laxity of eyelids

## Abstract

**Background**: Non-surgical procedures utilizing microcurrent radiofrequency (RF) represent a non-invasive option for patients experiencing skin laxity and loss of firmness due to aging, hormonal changes, or weight fluctuations. Such treatments benefit individuals seeking both preventive measures to delay visible aging and corrective approaches to improve existing skin laxity without invasive surgery. The Plasma IQ medical device generates microcurrent RF energy that produces controlled heating, leading to targeted tissue ablation and subsequent remodelling. This study aimed to evaluate the clinical efficacy of Plasma IQ in the treatment of skin laxity. **Materials and Methods**: Thirty patients presenting with facial skin laxity and photodamage underwent treatment with the Plasma IQ device. Clinical outcomes were assessed prior to the procedure and at 7, 21 and 90 days post-treatment. **Results**: RF treatment produced a measurable improvement in skin elasticity (+22.51%). The distance between the upper eyelid margin and the beginning of the eyelid fold increased by 2.01 mm (+46.94%) at day 21 and by 2.11 mm (+49.11%) at day 90. **Conclusions**: Microcurrent RF delivered by the Plasma IQ device is an effective non-invasive method for periocular skin rejuvenation. The treatment improves skin elasticity, reduces wrinkles around the eyes, and increases the visible distance between the upper eyelid margin and fold, thereby enhancing upper eyelid definition.

## 1. Introduction

The periocular region plays a pivotal role in social interaction and the expression of emotions, yet it is also among the earliest facial subunits to exhibit visible signs of aging. A youthful periocular appearance is defined by smooth skin with minimal laxity, an appropriate eyebrow position, and the absence of rhytides or pigmentation. With advancing age, however, complex structural and biochemical changes emerge, including redistribution and loss of adipose tissue, reduced dermal elasticity, deepening of wrinkles, skeletal remodelling, and alterations in muscle tone. While soft tissue descent was traditionally considered the primary driver of periocular aging, contemporary evidence emphasizes the multifactorial contributions of volume depletion, fat redistribution, and bone resorption [1,2,3]. In parallel, extrinsic factors such as chronic ultraviolet exposure, oxidative stress, and environmental insults accelerate collagen degradation, elastin fiber disorganization, and dermal thinning. Age-related reductions in fibroblast activity and extracellular matrix remodelling further diminish cutaneous resilience [4,5].

In response to the growing demand for safe and effective rejuvenation procedures, multiple non-invasive modalities have been developed to address periocular laxity, including fractional CO_2_ lasers, microneedling with radiofrequency, and high-intensity focused ultrasound [6]. Each technology has unique mechanisms of action, safety considerations, and limitations across different skin phototypes. Among emerging approaches, plasma skin regeneration (PSR) has attracted attention as a novel resurfacing technique. Unlike lasers or light-based devices, PSR is not chromophore dependent and does not vaporize tissue. Instead, it creates a controlled thermal effect while leaving a layer of desiccated epidermis intact, which serves as a biologic dressing that supports wound healing and accelerates recovery [7].

PSR has received FDA 510(k) clearance for the treatment of rhytides, superficial cutaneous lesions, actinic keratoses, viral papillomas, and seborrheic keratoses [8,9]. In addition, it has demonstrated clinical benefits in the management of dyschromia, photoaging, skin laxity, and acne scars. Its safety profile is favorable, and unlike CO_2_ laser resurfacing, PSR has not been associated with demarcation lines along aesthetic boundaries such as the perioral, periorbital, or mandibular regions [7]. Clinical evidence supports its efficacy for facial and periorbital rhytides, with applications extending to extrafacial areas including the neck, chest, and dorsal hands [10].

Plasma IQ, a CE-certified class IIa medical device, represents another plasma-based technology designed for aesthetic dermatology [11]. The device generates a plasma arc by ionizing atmospheric gas particles between the electrode tip and the skin surface. This arc induces sublimation of the superficial epidermis, directly converting solid tissue into gas and producing immediate, localized skin contraction with minimal thermal spread to adjacent structures. Treatment results in the formation of small carbon crusts, which typically detach within one week [12]. On a cellular level, plasma-induced microinjuries trigger physiological wound-healing responses, leading to gradual improvement in skin mechanical properties and clinical appearance [7,13]. The combination of immediate tissue contraction and long-term dermal remodelling underlies the rejuvenating effects of the treatment.

Despite the increasing popularity of plasma radiofrequency devices in aesthetic practice, high-quality clinical data evaluating their efficacy in periocular rejuvenation remain limited. The aim of this study was therefore to assess the clinical outcomes of a therapeutic protocol using microcurrent radiofrequency delivered via the Plasma IQ device for the treatment of periocular skin laxity.

## 2. Materials and Methods

### 2.1. Participants and Exclusion Criteria

Thirty patients (27 women and 3 men), aged 25–66 years (mean age 43.6 years), with periocular skin laxity and photodamage, and Fitzpatrick skin phototypes I–IV, were enrolled in the study. Patients underwent the treatment protocol described below.

Exclusion criteria included:

1. Use of oral and/or topical retinoids within the last 6 months.
2. Excessive tanning.
3. Active skin or connective tissue diseases associated with photosensitivity (e.g., systemic lupus erythematosus, porphyria cutanea tarda).
4. Active herpes simplex infection.
5. Use of drugs or photoreactive cosmetics within the last 6 months, including:
  - Tetracycline antibiotics
  - Immunosuppressive agents (e.g., corticosteroids and derivatives)
  - Anticoagulants (e.g., dipyridamole, coumarin derivatives)
  - Cosmetics containing thyme extract or herbal products such as St. John’s wort
6. Immunodeficiency disorders (including active HIV infection).
7. Fitzpatrick skin phototype VI.
8. Pregnancy (as a precaution).
9. Uncontrolled diabetes mellitus.
10. Previous cosmetic or aesthetic procedures in the treatment area (eligibility determined by the physician, depending on the procedure performed).
11. Acquired vitiligo or other disorders of melanin production (e.g., hypermelanosis).
12. Tattoos in the areas designated for treatment.
13. Use of anti-inflammatory medications.

Each patient signed an informed consent. The leading standard adopted in this study was ISO 14155:2020 [14]. The study was conducted in accordance with the Declaration of Helsinki and approved by the Ethics Committee of the Medical Chamber in Gdańsk, Poland (protocol code: 1/CMDRK/2020; 27 October 2020).

### 2.2. Treatment Protocol

On day 0, all patients were treated with the Plasma IQ medical device (Berger & Kraft Medical Sp. z o.o., Warsaw, Poland) on the upper eyelids, lower eyelids, and crow’s feet regions bilaterally. The procedure was performed using the manufacturer-defined LOW energy setting for aesthetic procedures. Energy was delivered manually in a point-by-point manner, with an inter-point spacing of approximately 1–2 mm, in a single treatment session conducted by trained operators according to a standardized protocol.

The total treatment time per session was approximately 25 min for the upper eyelids, 25 min for the lower eyelids, and 5–10 min for the lateral periocular (crow’s feet) region. All treatment areas were addressed bilaterally during the same session. The number of application points was determined by anatomical area and inter-point spacing rather than by a predefined number of points per cm^2^ [15].

#### Device Characteristics and Exposure Parameters

The Plasma IQ device (Berger & Kraft Medical Sp. z o.o., Raszyn, Poland) is a CE-certified class IIa medical device operating as a monopolar microcurrent radiofrequency (RF) generator with plasma-mediated energy delivery. When the active electrode tip is brought into close proximity with the skin surface, ionization of atmospheric gas particles occurs, resulting in the generation of a localized plasma arc without direct electrode–skin contact.

According to the manufacturer’s technical documentation, the RF generator operates at a frequency of 40 kHz, with a maximum power output of up to 5 W in continuous mode. In the LOW setting used in the present study, the generator output voltage is 650 V, while the voltage at the active electrode reaches approximately 950 V, enabling controlled plasma generation without the use of a neutral electrode [16].

Although the generator operates in continuous mode, tissue exposure is spatially and temporally segmented by manual point-by-point application of plasma energy. The duration of individual plasma applications is not user-adjustable and is not specified in milliseconds or seconds in the manufacturer’s technical documentation.

Each plasma application produces a discrete, highly localized superficial micro-injury. The resulting carbonized micro-lesions have a mean diameter of approximately 418 μm and a mean depth of approximately 99 μm, and typically detach spontaneously within approximately 7 days. These controlled micro-injuries are followed by gradual tissue remodeling and are clinically associated with improvements in skin firmness and elasticity over time.

Tissue-level power density and spatial electromagnetic field distribution were not independently quantified in this clinical study. Owing to plasma-mediated, highly localized energy delivery and internal generator control, these parameters cannot be reliably derived from voltage values alone and are therefore not reported.

### 2.3. Assessment Protocol

Day 0 (baseline): Before treatment, all patients underwent cutometric tests for skin elasticity and hydration. Three measurements were performed on the upper eyelids, lower eyelids, and crow’s feet regions. Anthropometric eyelid measurements (distance between upper eyelid margin and overlying skin fold) were also carried out.

Day 7: All patients underwent cutometric tests for skin elasticity and hydration, with three measurements performed on the upper eyelids, lower eyelids, and crow’s feet regions. Anthropometric eyelid distance measurements were also repeated.

Day 21: All patients underwent cutometric tests for skin elasticity and hydration, again with three measurements per site (upper eyelids, lower eyelids, crow’s feet). Anthropometric eyelid measurements were performed as at baseline.

Day 90: All patients underwent final cutometric tests for skin elasticity and hydration, with three measurements on the upper eyelids, lower eyelids, and crow’s feet areas. Anthropometric eyelid measurements were repeated in the same manner as at baseline.

### 2.4. Clinical Assessment

#### 2.4.1. Cutometric Evaluation

Cutometric tests were performed using the Courage + Khazaka Multi Skin Test Center MC1000 (Courage + Khazaka Electronic GmbH, Cologne, Germany) under standardized conditions of temperature (22–23 °C) and relative humidity (55–60%). Patients were prepared by removing makeup at least one hour before testing without alcohol-based cleansers. Measurements were conducted by trained personnel. Skin elasticity and hydration were measured on a 1–100 scale (1 = poorest result; 100 = best result). Three independent measurements were recorded for each site, and mean values were used for statistical analysis.

#### 2.4.2. Eyelid Skin Distance Evaluation

Anthropometric measurements were carried out using a caliper. All measurements were performed by the same investigator experienced in periocular assessment. Patients were positioned in the Camper plane, with eyes open and gaze fixed forward. The vertical distance between the upper eyelid margin and the inferior edge of the skin fold was measured along the midpupillary line. Each measurement was repeated three times, and mean values were used for statistical analysis.

#### 2.4.3. Healing Time Observation

Healing parameters were assessed by patient self-observation. Two variables were recorded: duration of post-procedural redness and swelling.

#### 2.4.4. Patient Satisfaction Evaluation

Patient satisfaction was assessed using a structured survey conducted 90 days after the procedure. Participants rated the overall aesthetic improvement of the treated periocular area on a five-point scale:

(1): no improvement.

(2): poor improvement.

(3): moderate improvement.

(4): good improvement.

(5): very good improvement.

Additionally, patients were asked to rate procedure-related pain on a five-point scale, ranging from 1 (painless) to 5 (very painful).

#### 2.4.5. Expert Evaluation

Independent evaluation was performed by a panel of seven dermatology and/or aesthetic medicine specialists, each with at least five years of clinical experience. The experts were presented with standardized photographs of patients taken at baseline and at 90 days post-procedure. They were blinded to the treatment details.

Assessment was carried out through an anonymous survey in which experts rated the overall aesthetic improvement of the treated area on the same five-point scale used for patient evaluation.

#### 2.4.6. Statistical Analysis

Statistical analysis of skin elasticity and hydration measurements was performed using IBM SPSS Statistics (version 28.0.1.0; 2021). Paired *t*-tests were used for mean comparisons.

1. Null hypothesis (H_0_): The procedure has no effect on the tested parameters.
2. Alternative hypothesis (H_1_): The procedure causes statistically significant changes in the tested parameters.

## 3. Results

### 3.1. Results of Clinical Assessment

#### 3.1.1. Cutometric Evaluation

1. Skin elasticity measurements

**Figure 1:**
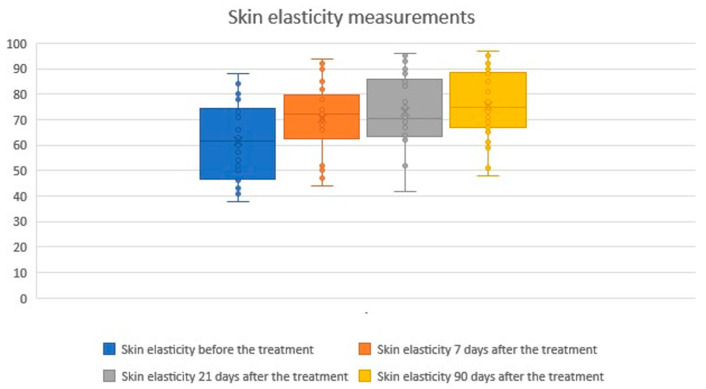
Skin elasticity measurements before and after treatment. Boxplots represent skin elasticity values measured at four time points: baseline (before treatment), 7 days after treatment, 21 days after treatment, and 90 days after treatment. An increase in both the median and overall distribution of skin elasticity was observed after treatment and maintained throughout the 90-day follow-up period. The × symbol represents the mean value, while the horizontal line inside the box indicates the median.

**Figure 2:**
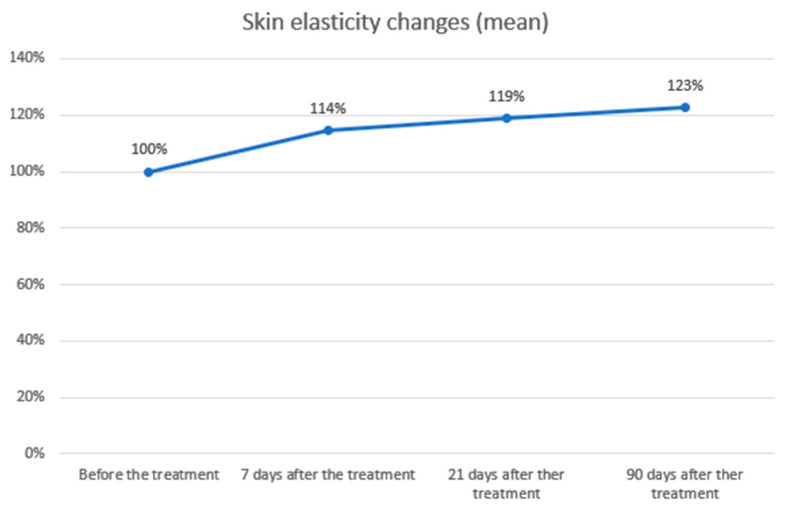
Skin elasticity changes over time. Skin elasticity was measured before treatment and at follow-up visits on days 7, 21, and 90. Baseline values were normalized to 100%. A progressive increase in elasticity was observed, rising to 114% after 7 days, 119% after 21 days, and 123% after 90 days. These results demonstrate a sustained and gradual improvement in skin elasticity following the procedure.

The study showed statistical significance and a positive correlation of the effect on changes in skin elasticity at 7, 21 and 90 days after the procedure. The effect is significant 7 days after the procedure and slightly improves over time. The average level of elasticity before the examination was 61.60, 7 days after the procedure 70.47 (+14.40%), 21 days after the procedure 73.13 (+18.72%) and 90 days after the procedure 75.47 (+22.51%). Statistical analysis and a graphical presentation of the results are presented in the tables and graphs below (Table 1; Figure 1 and Figure 2).

2. Skin hydration measurements

**Figure 3:**
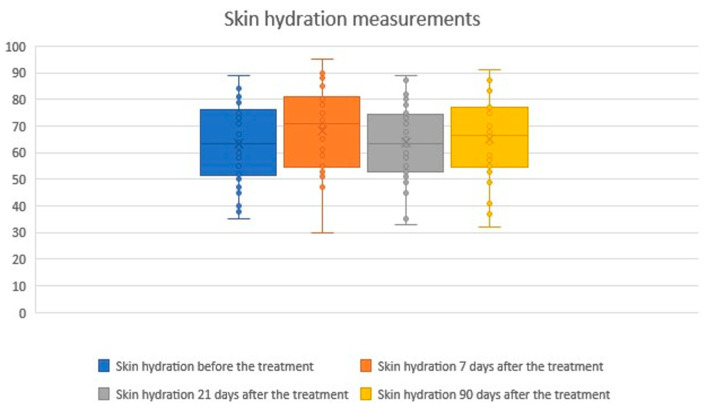
Skin hydration measurements before and after treatment. Boxplots illustrate skin hydration values assessed at baseline, 7 days, 21 days, and 90 days after treatment. A statistically significant increase in hydration was observed at day 7, while values at days 21 and 90 showed no significant differences compared with baseline. Overall, skin hydration remained relatively stable throughout the follow-up period. The × symbol represents the mean value, while the horizontal line inside the box indicates the median.

**Figure 4:**
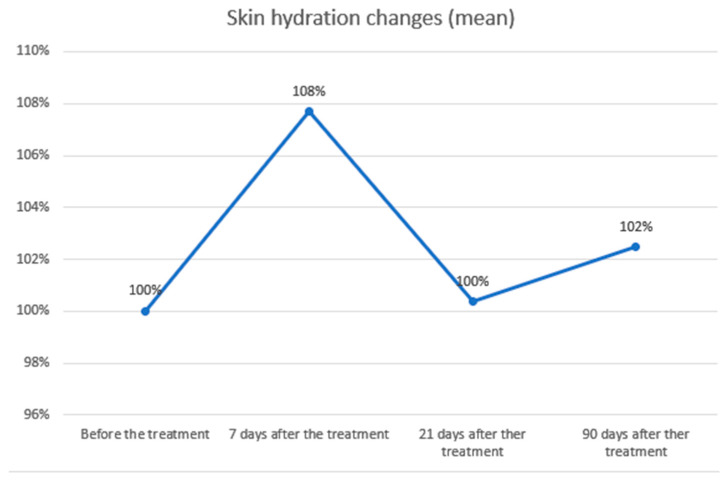
Skin hydration changes over time. Skin hydration levels were expressed as percentages relative to baseline (100%) and measured at 7, 21, and 90 days after treatment. A transient increase in hydration was observed at day 7 (108%), followed by a return to baseline levels at day 21 (100%) and a slight rise at day 90 (102%). Overall, hydration values fluctuated modestly and remained close to baseline, indicating minimal long-term changes.

The study demonstrated a statistically significant improvement and a positive correlation in skin hydration 7 days after the procedure, which is likely related to early stages of healing and mild tissue swelling. At 21 and 90 days post-treatment, skin hydration values showed positive correlations but without statistical significance. The mean skin hydration level at baseline was 63.40, increasing to 68.27 (+7.68%) at day 7, 63.63 (+0.37%) at day 21, and 64.97 (+2.47%) at day 90. A summary of the statistical analysis and graphical presentation of the results is provided in the tables and figures below (Table 2; Figure 3 and Figure 4).

#### 3.1.2. Eyelid Skin Distance Evaluation

**Figure 5:**
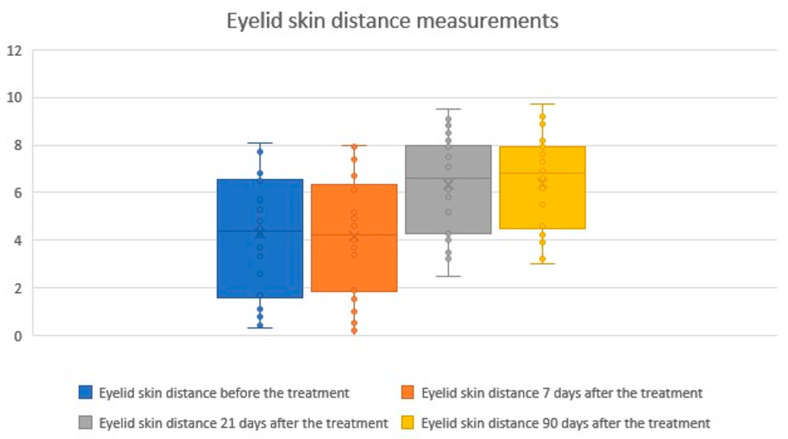
Eyelid skin distance measurements before and after treatment. Boxplots represent the distance between the eyelid margin and the overlying skin fold measured at baseline, 7 days, 21 days, and 90 days after treatment. No significant change was observed at day 7, whereas significant increases were detected at days 21 and 90. These findings indicate a progressive and sustained improvement in eyelid skin distance following the procedure. The × symbol represents the mean value, while the horizontal line inside the box indicates the median.

**Figure 6:**
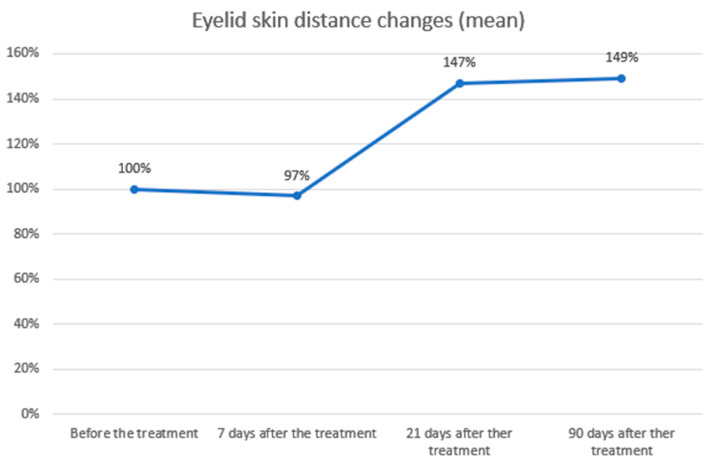
Eyelid skin distance changes over time. Eyelid skin distance was expressed as percentages relative to baseline (100%) and measured at 7, 21, and 90 days after treatment. A slight decrease was observed at day 7 (97%), followed by a marked increase at day 21 (147%) and a further improvement at day 90 (149%). These results demonstrate a significant and sustained increase in eyelid skin distance following the procedure.

The study demonstrated a statistically significant increase and a positive correlation in the distance between the eyelid margin and the overlying skin fold at 21 and 90 days after the procedure. The effect was significant at day 21 and showed further slight improvement by day 90. The mean eyelid skin distance at baseline was 4.30 mm, decreasing to 4.17 mm (−2.95%) at day 7 (a non-significant change, likely related to the early healing phase and transient swelling). At day 21, the distance increased to 6.31 mm (+46.94%) and further to 6.41 mm (+49.11%) at day 90. Statistical analyses and graphical representations of these findings are presented in the tables and figures below (Table 3; Figure 5 and Figure 6). Representative pre- and post-treatment photographs illustrating typical clinical changes are shown in Figure S1 (Supplementary Materials).

#### 3.1.3. Healing Time Observation

**Figure 7:**
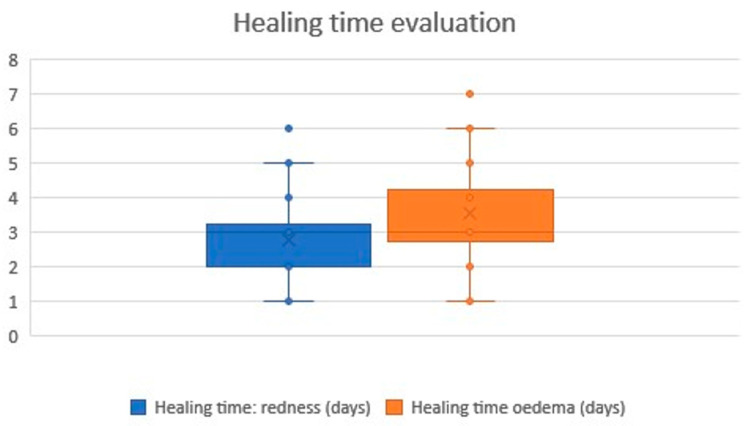
Healing time evaluation results. The chart illustrates the duration of post-treatment redness and oedema. Redness was reported by all participants and resolved within a mean of 2.77 days, while oedema lasted slightly longer, with a mean of 3.57 days. The × symbol represents the mean value, while the horizontal line inside the box indicates the median.

Healing following treatment with the Plasma IQ medical device was characterized by skin redness and local oedema. No other visible or subjective symptoms of healing were reported by the participants. All participants experienced skin redness, lasting between 1 and 6 days (mean 2.77 days), and local oedema, lasting between 1 and 7 days (mean 3.57 days). A graphical summary of these results is presented in the chart below (Figure 7).

#### 3.1.4. Patient’s Satisfaction Evaluation

Patients’ satisfaction with the procedure was generally high. In terms of general aesthetic improvement, 36.67% of patients declared very good improvement, 33.33% reported good improvement, 26.67% moderate improvement, and only 3.33% assessed the results as poor.

**Figure 8:**
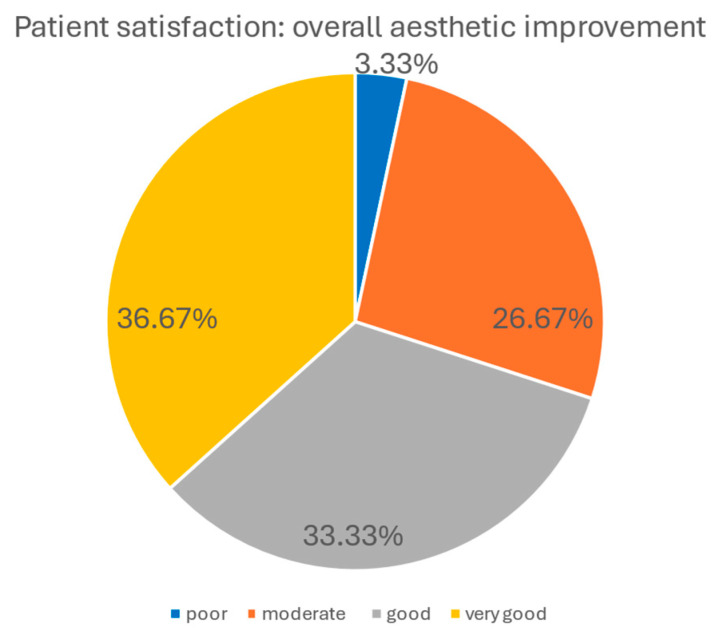
Patient’s satisfaction with general aesthetic improvement. Most patients reported moderate to very good improvement, while only 3.33% rated the results as poor.

**Figure 9:**
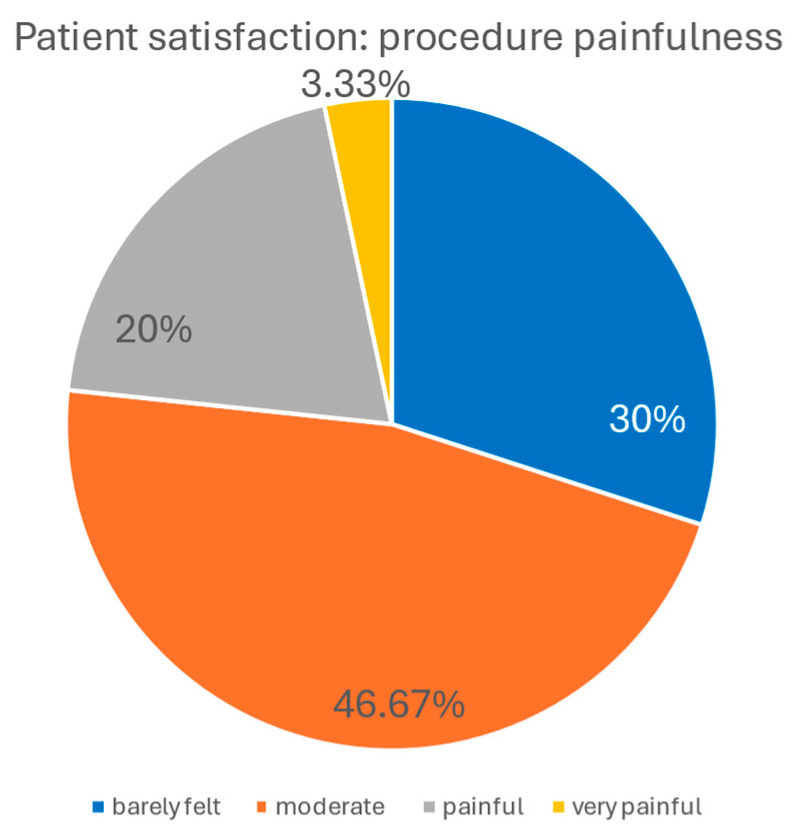
Patient’s evaluation of procedure’s painfulness. Nearly half of the patients (46.67%) rated the procedure as moderately painful, 30.00% as barely felt, 20.00% as painful, and only 3.33% as very painful.

Regarding the level of painfulness, 30.00% of patients described the procedure as “barely felt,” 46.67% as “moderately painful,” 20.00% as “painful,” and 3.33% as “very painful.” The graphical elaboration of the results is presented in the charts below (Figure 8 and Figure 9).

#### 3.1.5. Experts Evaluation

**Figure 10:**
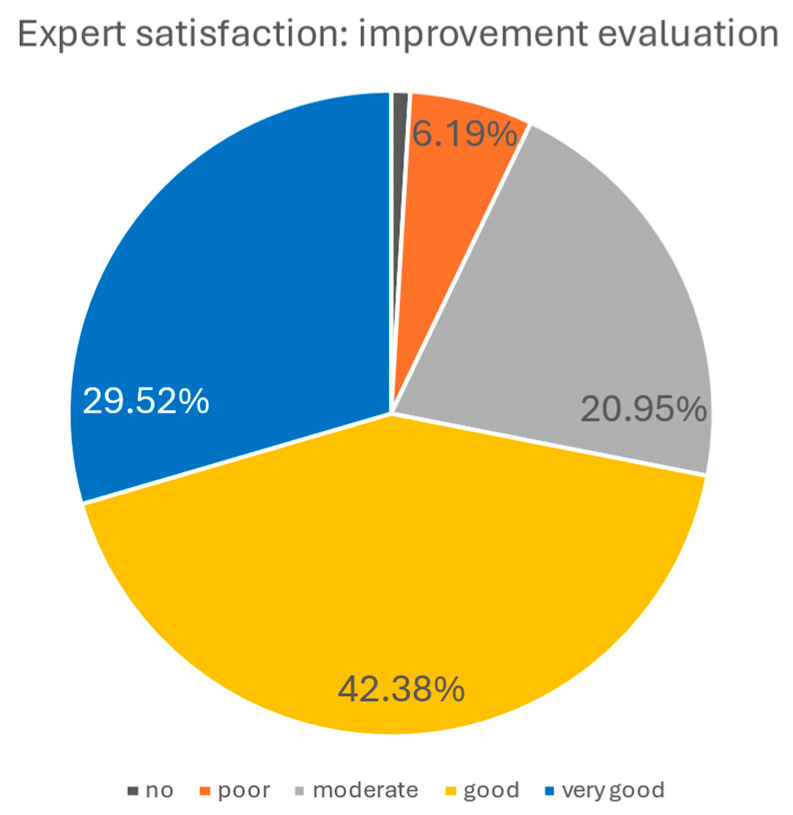
Experts’ satisfaction evaluation. Most experts reported good to very good improvement, while only a small proportion rated the results as poor or absent. The dark grey section of the pie chart has a value of 0.96 and represents no improvement.

Experts’ assessment of general aesthetic improvement following the procedure was, overall, positive. According to their evaluations, 29.52% indicated very good improvement, 42.38% good improvement, 20.95% moderate improvement, 6.19% poor improvement, and only 0.95% no improvement. The graphical representation of these results is shown in the chart below (Figure 10).

## 4. Discussion

This study provides comprehensive clinical evidence supporting the efficacy and safety of microcurrent radiofrequency treatment using the Plasma IQ device for rejuvenation of the eyelid and periorbital region. Our findings demonstrate measurable improvements in eyelid visibility, skin elasticity and structural skin quality parameters, alongside high levels of patient and expert satisfaction. These results underscore the clinical relevance of Plasma IQ as a minimally invasive modality for periorbital rejuvenation.

Radiofrequency microcurrent treatment with Plasma IQ led to a statistically significant increase in eyelid exposure, with the upper eyelid–skin fold distance rising by 46.95% at day 21 and 49.11% at day 90 (*p* < 0.01). A transient, non-significant decrease (−2.95%) observed at day 7 was most likely due to post-procedural swelling. This temporal distribution suggests a delayed biological response, such as skin remodelling, rather than immediate thermal shrinkage, in line with prior observations reported in the literature [11,17,18,19].

Although real-time temperature measurements and biochemical analyses were not performed in this study, plasma-mediated radiofrequency delivery is characterized by highly localized and transient tissue interactions rather than homogeneous bulk heating. Preclinical histological studies have shown that plasma/RF systems produce discrete microscopic zones of thermal effect comparable to conventional monopolar RF, without evidence of uncontrolled thermal spread [20,21]. In addition, plasma-mediated treatments are known to generate transient reactive oxygen and nitrogen species that act primarily as biological signaling mediators in wound healing and tissue remodeling pathways, and radiofrequency fields may modulate such responses through non-thermal mechanisms independent of measurable temperature increases [22,23,24]. Together, these mechanisms may contribute to the delayed and progressive clinical improvements observed after treatment.

Cutometric testing showed significant improvements in skin elasticity, with increases of 14.40% at day 7, 18.72% at day 21, and 22.51% at day 90 (*p* < 0.01). These findings are compatible with a dual-phase response described in the literature, potentially involving an early contraction effect followed by longer-term tissue remodelling [18,19]. Skin hydration improved slightly (7.68%, *p* < 0.01) at day 7, probably due to transient edema, but no significant changes were noted at later points. Combining Plasma IQ with hyaluronic acid (HA) injections may further enhance hydration and overall skin quality, as synergistic effects have been observed with other RF- or IR-based therapies combined with HA [25].

Patient-reported outcomes were in agreement with independent expert evaluations and aligned with objective measurements. At 90 days post-treatment, approximately 70% of patients rated the results as very good or good, while over 71% of expert assessments provided comparable ratings. This convergence underscores the perceptible and clinically meaningful improvements achieved with the procedure. However, the moderate ratings reported by 26.7% of patients and 20.9% of experts indicate interindividual variability in treatment response and suggest that outcomes may be further optimized through tailored treatment protocols.

The treatment was well tolerated. Pain levels were reported as “barely felt” or “moderately painful” by 76.7% of patients. The most common side effects were transient erythema (1–6 days, mean 2.77 days) and edema (1–7 days, mean 3.57 days). No severe adverse effects occurred, underscoring the favorable tolerability profile of Plasma IQ compared with more invasive ablative modalities [18].

Compared with ablative laser technologies (e.g., CO_2_ or Er:YAG), Plasma IQ produces more modest epidermal changes but demonstrates robust efficacy in dermal tightening with minimal downtime [26,27]. This balance of safety and effectiveness makes it particularly well-suited for the delicate periorbital region, where tolerance for adverse effects is low. Importantly, the results presented here stem from a single treatment; repeated sessions at 8–12-week intervals may further enhance and prolong outcomes.

The main limitations of this study include the small sample size, single-arm design, and 90-day follow-up. Due to the exploratory nature and limited cohort, normality testing and nonparametric sensitivity analyses were not performed; future larger studies should incorporate these methods as well as comparative designs versus other non-ablative modalities (e.g., HIFU, fractional RF). Although device parameters were reported according to manufacturer data, tissue-level power density and electromagnetic field distribution were not independently measured. Real-time temperature monitoring and biochemical analyses of oxidative stress or ROS/RNS markers were also not conducted. Future studies should include standardized thermography or intradermal temperature probes alongside biochemical assays of redox and inflammatory markers to better characterize treatment effects. Eyelid distance was measured using caliper-based anthropometry; digital photogrammetry may improve reproducibility in future trials.

Plasma IQ microcurrent radiofrequency treatment effectively improves eyelid visibility and skin elasticity, with results supported by objective measurements, patient surveys, and expert assessment. The procedure was safe, well tolerated, and associated only with transient erythema and swelling. These findings highlight Plasma IQ as a valuable, minimally invasive option for periorbital skin rejuvenation. Further studies with larger cohorts, repeated treatment protocols, and extended follow-up are warranted to optimize outcomes and confirm the long-term benefits of this modality.

## Figures and Tables

**Table 1:** Changes in skin elasticity before and after treatment.

Time Point	Mean ± SD	Δ vs. Baseline (Mean ± SD)	95% CI of Difference	*p*-Value
Baseline	61.60 ± 15.14	–	–	–
Day 7	70.47 ± 14.20	+8.87 ± 9.00	−12.23 to −5.39	<0.001
Day 21	73.13 ± 13.74	+11.53 ± 6.28	−13.88 to −9.19	<0.001
Day 90	75.47 ± 13.26	+13.87 ± 6.72	−16.38 to −11.36	<0.001

Mean ± SD values of skin elasticity are shown for baseline, day 7, day 21, and day 90 (N = 30). Differences vs. baseline, 95% confidence intervals, and *p*-values (paired *t*-test) are reported. All post-treatment measurements demonstrated significant improvements compared with baseline (*p* < 0.001).

**Table 2:** Skin hydration before and after treatment.

Time Point	Mean ± SD	Δ vs. Baseline (Mean ± SD)	95% CI of Difference	*p*-Value
Baseline	63.40 ± 14.79	–	–	–
Day 7	68.27 ± 15.83	+4.87 ± 6.22	−7.19 to −2.54	<0.001
Day 21	63.63 ± 14.40	+0.23 ± 3.65	−1.60 to 1.13	0.73
Day 90	64.97 ± 15.39	+1.57 ± 8.11	−4.59 to 1.46	0.30

Mean ± SD values of skin hydration are shown for baseline, day 7, day 21, and day 90 (N = 30). Differences vs. baseline, 95% confidence intervals, and *p*-values (paired *t*-test) are reported. A statistically significant increase in skin hydration was observed at day 7, while changes at days 21 and 90 were not significant.

**Table 3:** Changes in eyelid skin distance before and after treatment.

Time Point	Mean ± SD	Δ vs. Baseline (Mean ± SD)	95% CI of Difference	*p*-Value
Baseline	4.30 ± 2.55	–	–	–
Day 7	4.17 ± 2.47	+0.13 ± 0.36	−0.01 to 0.26	0.07
Day 21	6.31 ± 1.98	+2.02 ± 0.79	−2.31 to −1.72	<0.001
Day 90	6.41 ± 1.94	+2.11 ± 0.85	−2.43 to −1.79	<0.001

Mean ± SD values of the distance between the eyelid margin and the skin fold are shown for baseline, day 7, day 21, and day 90 (N = 30). Differences vs. baseline, 95% confidence intervals, and *p*-values (paired *t*-test) are reported. No significant change was observed at day 7, whereas statistically significant increases were detected at days 21 and 90, indicating a sustained improvement over time.

## Data Availability

The data presented in this study are available upon request from the corresponding author. The data are not publicly available due to privacy restrictions.

## Supplementary Materials

The following supporting information can be downloaded at https://www.mdpi.com/article/10.3390/biomedicines14030679/s1, Figure S1. Representative clinical photographs before and after treatment.
