# Supplementary material for: Clinical and Instrument-Based Evaluation of Plasma IQ Microcurrent Radiofrequency for Periorbital Skin Rejuvenation
Source: Biomedicines. 2026 Mar 16;14(3):679. doi: 10.3390/biomedicines14030679 (PMC13024019; doi:10.3390/biomedicines14030679)
Supplement: Supplementary file 1 [file biomedicines-14-00679-s001.zip › biomedicines-4177059-supplementary.pdf]

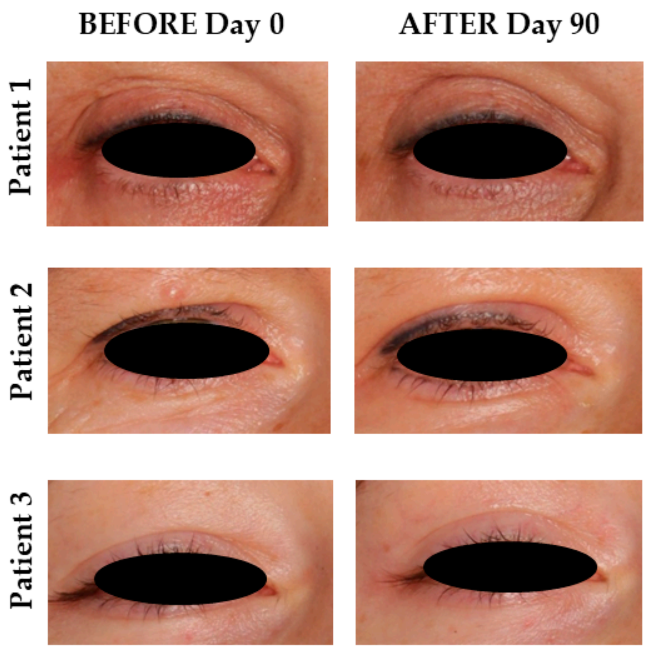

Figure S1. Representative clinical photographs before and after treatment.

Representative clinical photographs of three patients obtained at baseline (Day 0) and at Day 90 following a single Plasma IQ treatment. Images were captured with a forward gaze and relaxed facial expression and are presented to qualitatively illustrate changes in periocular skin appearance. Photographs were anonymized by cropping to the periocular region.
